# Contrast medium dose optimization in the era of multi-energy CT

**DOI:** 10.1007/s11604-025-01823-4

**Published:** 2025-06-24

**Authors:** Yasunori Nagayama, Takeshi Nakaura, Kazuo Awai, Kazuhiro Katahira, Satoru Takahashi, Noriko Oyama-Manabe, Satoshi Goshima, Yasuyuki Kobayashi, Takamichi Murakami, Toshinori Hirai, Masahiro Jinzaki

**Affiliations:** 1 Department of Diagnostic Radiology, Graduate School of Medical Sciences, Kumamoto University, 1-1-1, Honjo, Chuo-ku, Kumamoto, 860-8556 Japan; 2 Department of Diagnostic Radiology, Graduate School of Biomedical and Health Science, Hiroshima University, 1-2-3 Kasumi, Minamiku, Hiroshima, 734-8551 Japan; 3 Department of Diagnostic Radiology, Kumamoto Chuo Hospital, 1-5-1 Taishima, Minami-ku, Kumamoto, 862-0965 Japan; 4 Imaging Research Center, Takatsuki General Hospital, 1-3-13 Kosobe-cho, Takatsuki, Osaka 569-1192 Japan; 5 Department of Radiology, Jichi Medical University Saitama Medical Center, 1-847 Amanuma-cho, Omiya-ku, Saitama, 330-8503 Japan; 6 Department of Radiology, Hamamatsu University School of Medicine, 1-20-1 Handayama, Chuo-ku, Hamamatsu, Shizuoka, 431-3192 Japan; 7 Department of Medical Information and Communication Technology Research, Graduate School of Medicine, St. Marianna Univereity School of Medicine, 2-16-1 Sugao, Miyamae-ku, Kawasaki, Kanagawa 216-8511 Japan; 8 Department of Radiology, Kobe University Graduate School of Medicine, 7-5-2 Kusunoki-cho, Chuo-ku, Kobe, 650-0017 Japan; 9 Department of Radiology, Keio University School of Medicine, 35 Shinanomachi, Shinjuku-ku, Tokyo, 160-8582 Japan

**Keywords:** Multi-energy CT, Dual-energy CT, Photon-counting detector CT, Iodinated contrast medium, Iodine k-edge, Low X-ray energy

## Abstract

With the increasing use of contrast-enhanced CT, optimizing the iodinated contrast medium (ICM) dose while maintaining diagnostically adequate image quality is essential to mitigate potential adverse effects on patients, the environment, and public health, as well as to reduce medical costs and address potential supply shortages. Multi-energy CT technologies including dual-energy CT and photon-counting detector CT enable data acquisition at multiple energy spectra, allowing for material characterization beyond the capabilities of conventional single-energy CT. Recent technical advancements and the growing adoption of these technologies in clinical practice have enhanced patient care across various diagnostic tasks. Among the spectral-based imaging options offered by multi-energy CT, virtual monoenergetic imaging holds significant promise for substantial ICM dose reduction due to the drastic improvement in iodine contrast at lower energy levels. This article aims to provide an overview of multi-energy CT technology and its utility for ICM dose optimization across various clinical indications, while also discussing current issues and related topics.

## Introduction

Contrast-enhanced CT utilizing the iodinated contrast medium (ICM) is well established diagnostic tool for evaluating various medical conditions. Owing to its excellent diagnostic performance, wide clinical applicability, and easy accessibility, the number of contrast-enhanced CT examinations has been drastically increased. Currently, more than 10 million liters of ICM are used for contrast-enhanced CT every year worldwide [1]. This trend is accompanied by the increase in iodine exposure to the patients and the environments, and there is a growing demand to optimize the ICM dose to mitigate the associated possible adverse effects.

With regard to the patient safety, the most important issue on large amount of ICM administration is postcontrast acute kidney injury (PC-AKI), which has been reported as a risk factor for dialysis, renal failure, and death [2]. Recent large-scale propensity matched studies have shown that causal relationships between intravenous ICM administration and the development of PC-AKI have been overestimated in the past [3]. Although the true risk of PC-AKI with contrast-enhanced CT is still a matter of debate, the caution should be exercised in patients with severe renal disfunction, especially in the presence of comorbid risk factors such as hypertension and diabetes [4–9]. Given the reported possible relationship between the amount of ICM and the risk of PC-AKI developments, optimizing the ICM dose as low as possible to maintain the diagnostic image quality is desirable.

The optimization of ICM dose has also implications for broader healthcare systems and environmental sustainability. The large annual volume of CT scans worldwide highlights the potential of ICM dose reduction in individual examinations for substantial healthcare cost savings at both institutional and population levels [10]. This is particularly relevant in the context of increasing healthcare costs and the need for more efficient resource allocation. Regarding the environmental impact, the discharge of iodine excreted by patients into aquatic ecosystems and the water cycle can lead to contamination of drinking water, posing risks to both environmental and public health [11]. ICM are highly water-soluble and metabolically stable, making them difficult to remove during conventional water purification [11]. Residual ICM in water sources used for drinking water production may lead to the formation of iodinated disinfection by-products, which are known to be cytotoxic and genotoxic [11]. In this context, there is growing concern regarding potential long-term health risks associated with chronic exposure through drinking water. Minimizing ICM doses may help mitigate these environmental and public health risks, thereby promoting more sustainable and responsible medical practices. Furthermore, recently encountered disruptions in the global supply chain of ICM have underscored the importance of optimizing ICM use as a crucial strategy for ensuring the continuity of imaging services during supply shortages [12].

Several established approaches exist for lowering ICM doses, including scan timing optimizations using the bolus-tracking technique [13], administering a saline flush immediately after ICM administration [14], individualizing ICM doses based on body size [15–17], employing high-pitch or wide-coverage detector image acquisitions [18, 19], and utilizing low tube voltage scanning [20, 21]. Additionally, recent technological advancements have positioned multi-energy CT as a promising method for improving iodine contrast and reducing the ICM doses needed to achieve diagnostic image quality, particularly through the use of virtual monoenergetic imaging (VMI). This article aims to provide an overview of multi-energy CT technology and its utility for ICM dose optimization across various clinical indications, while also discussing current issues and related topics.

## Technical principles of multi-energy CT

**Fig. 1:**
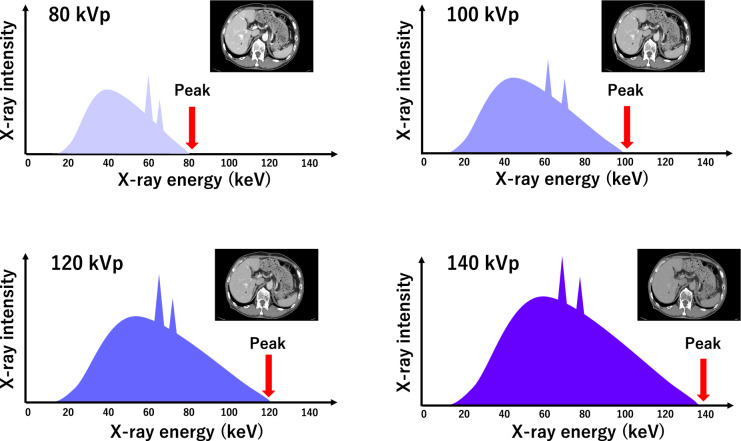
A polyenergetic X-ray beam is composed of photons with a wide range of energies. The"kVp"defines the upper limit of the polychromatic X-ray beam energy. In conventional single-energy CT, only one kVp image dataset per scan is available for diagnostic evaluation

X-ray tubes used in medical imaging produce polyenergetic beams composed of a wide range of photon energies (keV), with the upper limit of this energy spectrum defined by the kilovolt peak (kVp) (Fig. 1). SECT scanners generate CT images by calculating the linear attenuation coefficient of scanned materials at a given effective photon energy level, which is then normalized by the linear attenuation coefficient of water and displayed as Hounsfield Units (HU). Conventional SECT scans are usually performed at a tube voltage of 120 kVp.

The linear attenuation coefficient of a material is influenced by its chemical composition, specifically its effective atomic number (Zeff) and its mass density (ρ), as well as the photon energy of the X-ray beam. In SECT, materials with different compositions can exhibit similar CT attenuation values if one material has a higher effective atomic number and the other has a higher mass density. For example, the iodine (Zeff = 53) can exhibit the same attenuation as calcium (Zeff = 20) at a specific mass density and photon energy level. This overlap in attenuation values makes it impossible to distinguish and quantify these different compositions, presenting a significant limitation of conventional SECT. Although the tube voltage can be optimized in SECT according to the diagnostic task and the patient's body size, only one kVp image dataset per scan is available for diagnostic evaluation.

**Fig. 2:**
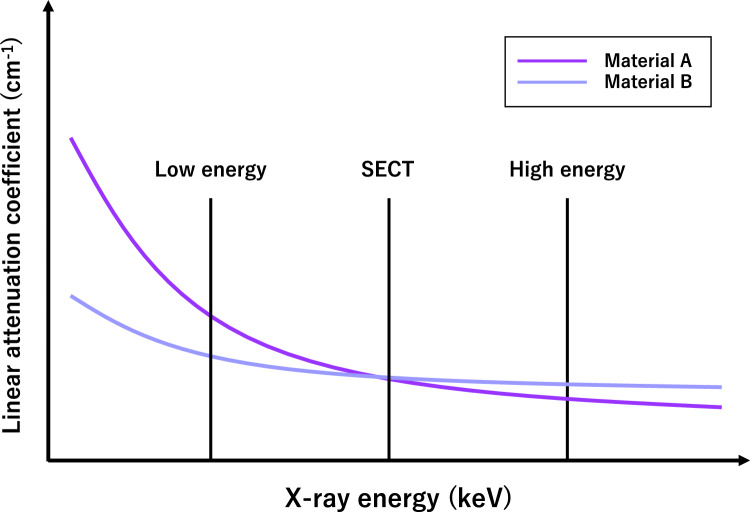
In SECT, different materials such as iodine and calcium can exhibit the same attenuation, making them difficult to distinguish. With multi-energy CT, these materials can be distinguished and quantified based on their different attenuation properties at various energy levels

Multi-Energy CT, which includes both dual-energy CT (DECT) and photon-counting detector CT (PCD-CT), addresses the limitations of SECT by acquiring attenuation data at two or more polyenergetic X-ray levels [22–25]. By using attenuation information obtained at different X-ray energy spectra, tissue composition can be differentiated and quantified based on the distinct X-ray attenuation profiles at different energies (Fig. 2). Given that CT attenuation is primarily due to photoelectric and Compton effects that depend on the Zeff and ρ, respectively, the attenuation properties of any materials can be modeled by these combinations. Multi-energy CT can provide various type of spectral-based image sets including iodine density maps, VMI, virtual non-contrast images, and virtual non-calcium images.

## Multi-energy CT systems

The multi-energy CT systems currently utilized in clinical practice include tube-based approach (e.g., dual-source DECT and fast kV switching DECT) or detector-based approach (dual-layer detector DECT and PCD-CT).

### Tube-based dual-energy CT

In dual-source DECT, two X-ray tubes and detectors are arranged approximately orthogonally, with one tube operating at low energy (70–100 kVp) and the other at high energy (140 or 150 kVp). A tin filter can be applied to the high-energy beam to improve spectral separation, with material decomposition being based on three materials (iodine, soft tissue, and fat or water). Due to temporal and spatial misregistration between low- and high-energy data, only image-domain spectral reconstruction is feasible, which can lead to less accurate beam-hardening correction [22–25]. Fast kV switching CT employs a single X-ray tube that rapidly alternates between low (80 kVp) and high (135 or 140 kVp) tube voltages during a single rotation, yielding near-synchronous data acquisition [22–25]. Although slight temporal and spatial misregistration requires interpolation to prevent streak artifacts, this technique allows projection-based material decomposition, reducing beam-hardening artifacts [22–25]. Both dual-source and fast kV switching CT scanners require prospective selection of the dual-energy mode before each examination, which can complicate clinical workflows.

### Detector-based dual-energy CT

Dual-layer detector CT uses a single X-ray source and a detector with two layers that separately absorb low- and high-energy photons, allowing perfectly registered dual-energy data acquisition [22–25]. No misregistration is desirable for improving the image quality and the accuracy of material decomposition. This is particularly relevant in motion-prone structures such as cardiac, lung, and intestinal regions, where physiological motion can lead to temporal and spatial misregistration between energies. This system supports projection-based material decomposition without the need for prior patient selection or workflow changes, as all acquisitions are automatically performed in multi-energy mode. Additionally, conventional polyenergetic images (e.g., 100, 120, or 140 kVp) can be generated simultaneously. The possible limitation is that the relatively large overlap between high and low energies can reduce the accuracy of material decomposition.

### Photon-counting detector CT

Recently, PCD-CT systems have been clinically introduced or are currently under development by manufacturers. PCD-CT systems are equipped with semiconductor detectors that can directly convert incoming photons into electrical signals, eliminating the need for converting X-rays into light and septa to separate individual detector elements. The details of this technology have been thoroughly documented in previous literatures [22, 23, 25, 26]. Compared to energy-integrating detector (EID) CT systems, PCD-CT offers several advantages, including higher spatial and contrast resolution, improved dose efficiency, and better energy separation. With reduced electronic image noise, PCD-CT may provide spectral imaging with improved image quality compared to EID-DECT systems.

## Low kVp scanning and low keV virtual monoenergetic image

Iodine attenuation increases as X-ray energy approaches the k-edge of iodine (33.2 keV) due to the enhanced photoelectric effect [27, 28]. Therefore, lowering the tube voltage on SECT scanning can improve iodine contrast and reduce the required ICM dose while maintaining adequate contrast of iodinated structures. Reducing the tube voltage from 120 to 100 kVp and 80 kVp increases the iodine contrast at approximately 25% and 70%, respectively. This means that ICM dose can be reduced by approximately 20% and 40% at 100 kVp and 80 kVp, respectively, while preserving vessel and organ contrast. Although the image noise at low kVp scanning increases due to reduced X-ray photons, the combined use of high tube currents with iterative reconstruction or deep-learning reconstruction techniques can mitigate the increased image noise, thereby providing diagnostic image quality even at lower radiation doses [29–34]. However, low tube voltage scanning lacks the flexibility to retrospectively optimize iodine contrast based on specific clinical requirements or the organ of interest after the images are acquired. Moreover, even at the lowest tube voltage settings, the effective X-ray energy remains higher than iodine’s k-edge, which may unexpectedly result in insufficient iodine contrast under certain conditions. Despite these possible limitations, low kVp scanning is well established and highly recommended technique for optimizing iodine dose particularly when only conventional SECT scanners are available.

**Fig. 3:**
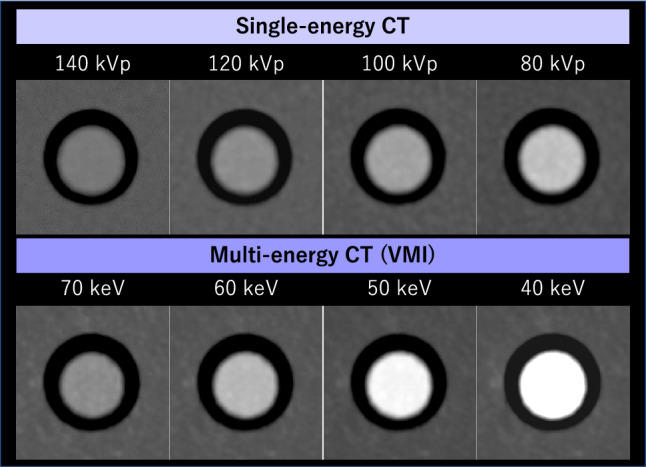
The iodine contrast of polyenergetic images ranging from 80 to 140 kVp in single-energy CT and VMI ranging from 40 to 70 keV in multi-energy CT (dual-layer detector scanner). VMI levels below 60 keV yield higher iodine contrast than polyenergetic 80 kVp images

Multi-energy CT can provide a continuum of VMIs at wide energy ranges, which replicates the images that would be obtained with a monoenergetic X-ray based on the results of material decomposition [22–26, 35, 36]. VMI at approximately 70 keV provides equivalent attenuation to conventional polyenergetic 120 kVp images, with reduced artifacts and noise [35]. Lower keV images improve iodine contrast due to greater photoelectric effect. Of note, the iodine contrast in the lowest keV range (e.g. 40–50 keV) can be more pronounced than that achieved with low kVp scanning (Fig. 3) [37–39], potentially allowing for a greater reduction in ICM dose when image noise and spatial resolution remain diagnostically acceptable level. VMI at 50 and 40 keV provide approximately 2.5-fold and 3.3-fold higher iodine contrast, respectively, compared to 120-kVp images [40]. Therefore, the iodine dose can be theoretically reduced by 60% and 70%, respectively, while maintaining comparable contrast enhancement. Furthermore, iodine contrast can be retrospectively optimized according to the specific requirements of each examination and organ of interest, even after image acquisition, effectively compensating for any unexpected degradation in contrast enhancement.

## Image noise reduction of low keV VMI

The image noise in lower energy VMIs is amplified compared to intermediate energy VMIs (e.g., 70 keV), which degrades the benefits of improved iodine contrast, especially in low-contrast diagnostic tasks susceptible to image noise such as abdominal CT. Earlier studies using initial DECT systems reported that the image quality was significantly compromised at lower energy ranges, despite the increased iodine contrast [41, 42]. However, recent advancements in multi-energy CT technology have implemented various noise reduction techniques that have considerably improved the clinical utility of low keV VMIs. For example, dual-source DECT now incorporates a noise reduction algorithm (Mono +) that merges the high signal at lower energy datasets with the noise reduction from higher energy datasets by using frequency-split technique [43, 44]. Dual-layer detector CT achieved minimal noise variability among entire energies by incorporating an anti-correlated noise reduction algorithm in the projection domain, maximizing the utility of low keV VMI [45, 46]. Fast kV switching CT systems have addressed increased noise with the introduction of deep learning-based technologies [47–49]. PCD-CT may reduce the image noise of low keV VMI compared to that of EID-DECT even at lower radiation doses because of its inherent dose efficiency. These advancements in multi-energy techniques facilitate reductions in ICM doses while maintaining or even improving diagnostic image quality. However, given the considerable variations in image properties across different multi-energy scanners [50–53] and the impact of body size on image quality [54], the optimal ICM dose protocol should be tailored to each specific CT system, patient size, and clinical indications. In addition, most previous studies have focused on evaluating the image quality of low ICM dose protocols rather than their diagnostic performance, underscoring the need for continuous and rigorous further investigations.

## Review of ICM dose reduction protocols with multi-energy CT

### Cerebral CT angiography

**Fig. 4:**
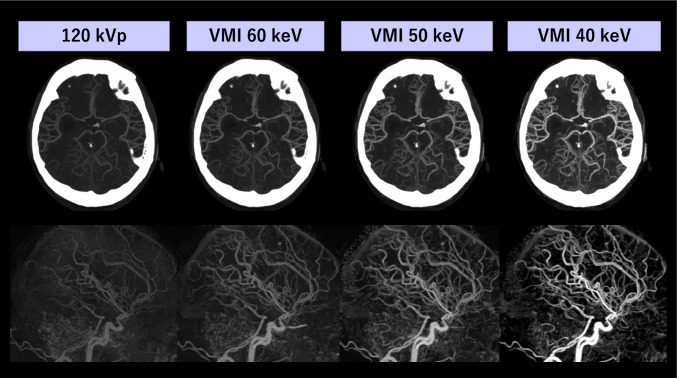
A 40-year-old man with subarachnoid hemorrhage underwent cranial CTA for treatment planning using dual-layer detector scanner. An ICM dose of 25 mL (370 mgI/mL, diluted with 25 mL of normal saline) was injected over 10 s. Conventional 120-kVp imaging resulted in poor arterial enhancement. Arterial attenuation gradually increased as VMI energy decreased, with excellent visualization of intracranial arteries observed at 40 keV VMI. The short ICM injection duration also helped to minimize venous contamination at the cavernous sinus

Cerebral CT angiography (CTA) provides the critical information for the managements of cerebral vascular disorders such as vessel occlusion, stenoses, and aneurism rupture for rapid decision making. A minimization of the ICM dose in this diagnostic task may be desirable particularly for emergency settings, because additional intra-arterial ICM administrations would be required in subsequent invasive angiography and endovascular therapy. As shown in Fig. 4, the visibility of cerebral arteries in low iodine dose cerebral CTA gradually improves as the energy level decreases, with maximum conspicuity observed at 40 keV. Zhao et al. demonstrated that 40 keV VMI from dual-source DECT reconstructed with Mono + yielded the image quality comparable to 120-kVp equivalent images even at 27% reduced iodine dose [55]. Zhou et al. also showed that 55 keV VMI from DLCT yielded non-inferior image quality even at 44% lower radiation and 59% lower ICM doses compared with conventional 120-kVp protocol [56]. In cerebral CTA, administration of ICM at short duration with high flow rate and scanning at optimal timing are required to minimize the venous contamination at the cavernous sinus that makes cerebral artery evaluation difficult [57]. In this context, Fransson et al. demonstrated that low keV VMIs with 50% ICM dose can retrospectively salvage non-diagnostic examinations even for suboptimal scanning timing [58]. The improved image quality of PCD CT relative to EID CT is also demonstrated for carotid and intracranial CTA [59], which may be translatable to ICM and radiation dose reduction. In addition to ICM reduction, the automated bone removal technique with material decomposition improves the visualization of arteries around the skull, which can reduce the total radiation dose by eliminating pre-contrast image acquisition for bone subtraction [60]. Moreover, low keV VMI enhances the characterization of carotid atherosclerotic plaque, such as lipid-rich necrotic cores, serving as markers of vulnerable plaques and increased ischemia risk [61].

### CT pulmonary angiography

**Fig. 5:**
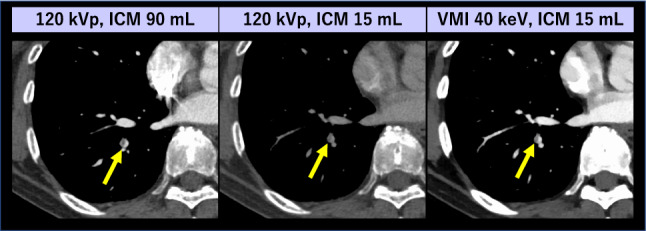
A 68-year-old woman with ovarian cancer and pulmonary embolism underwent two CTPA scans. The initial CTPA (left) was performed with a standard protocol (ICM 90 mL [370 mgI/mL]) using single-energy CT scanner. The follow-up CTPA (middle and right) was performed with a reduced ICM protocol (ICM 15 mL [370 mgI/mL]) using dual-layer detector scanner. In lower ICM dose protocol, a small pulmonary embolism (arrows) is barely visible at 120 kVp but easily detectable at VMI 40 keV, similar to the visibility in a standard ICM dose 120-kVp image

**Fig. 6:**
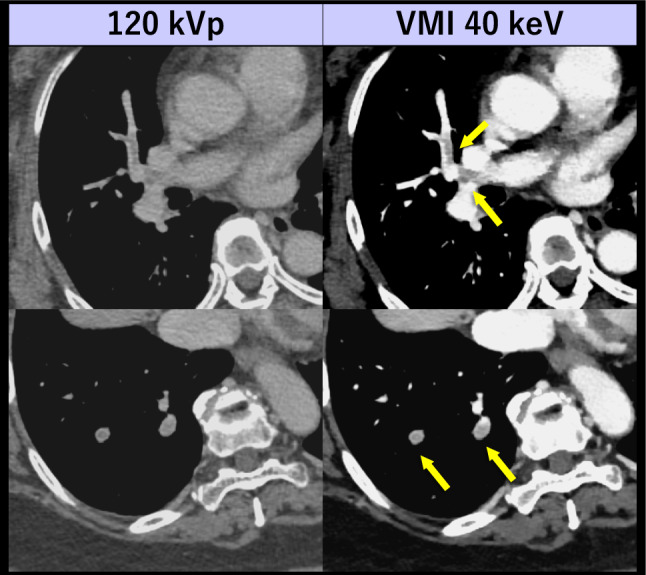
A 66-year-old woman with breast cancer who underwent portal venous phase CT using dual-layer detector scanner. Incidental PEs (arrows), which may be missed on 120 kVp images due to suboptimal contrast enhancement in the pulmonary arteries, are confidently detectable on VMI 40 keV without the need for dedicated CTPA

CT pulmonary angiography (CTPA) is the reference standard for diagnosing pulmonary embolism (PE). Previous studies have indicated that the low-keV VMI from DECT enabled up to 80% reduction of ICM dose while maintaining the diagnostic pulmonary artery (PA) enhancement and image quality [62, 63] (Fig. 5). With recently introduced PCD-CT, the image quality of CTPA can be improved by utilizing 40–50 keV VMI [64]. Pannenbecker et al. indicated that low keV VMI from PCD-CT provided better image quality than dual-source EID DECT for CTPA even at half ICM and radiation doses [65]. Even with standard ICM dose, suboptimal PA opacification due to physiological or technical factors can be retrospectively optimized with VMI. The transient interruption of contrast, caused by an increase in unopacified blood return from the inferior vena cava during deep inspiration, can reduce PA opacification. This insufficient PA opacification can be retrospectively compensated by lowering the keV in VMI, thereby improving diagnostic accuracy in detecting PE potentially and potentially avoiding the need for re-examinations [66]. Low-keV VMI also facilitates the detection of incidental PE with venous phase CT performed for other clinical indications [67], mitigating the necessity for dedicated CTPA scanning involving the additional radiation and ICM exposure (Fig. 6).

### Coronary CTA

**Fig. 7:**
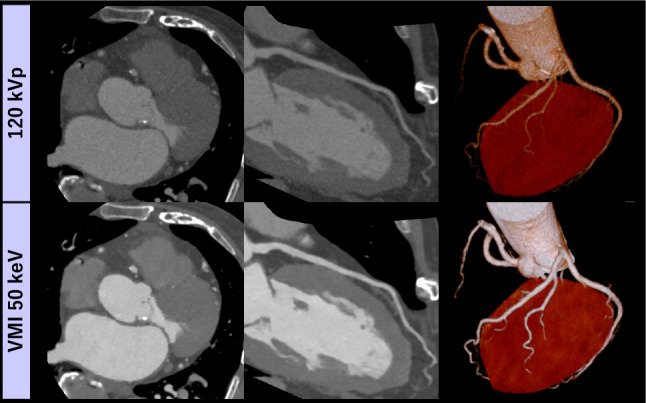
An 85-year-old man (BMI 23 kg/m^2^) presenting with chest pain underwent coronary CTA using a low ICM dose (19 mL, 370 mgI/mL) on dual-layer detector scanner. The low keV VMI reconstruction demonstrated superior iodine contrast and enhanced visualization of small and peripheral coronary arteries compared to conventional 120 kVp images. Image courtesy of Kumamoto Chuo Hospital

**Fig. 8:**
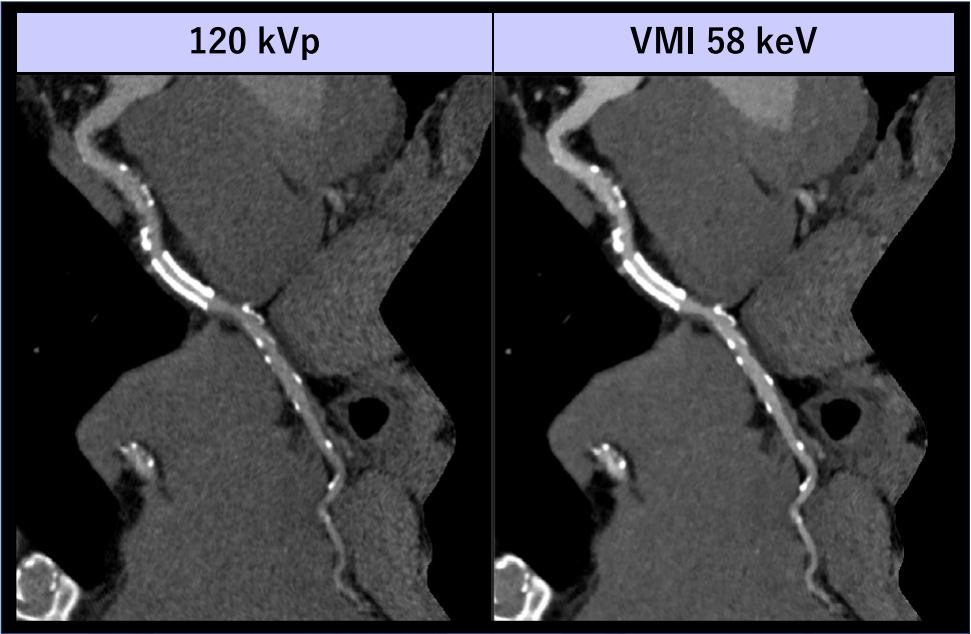
A 70-year-old man (BMI 27 kg/m^2^) post-PCI underwent coronary CTA using dual-layer detector scanner with a low dose of ICM (21 mL, 350 mgI/mL). The low keV VMI provided sufficient coronary enhancement while preserving the visibility of in-stent patency. Image courtesy of Minamino Cardiovascular Hospital

The risk factors of PC-AKI such as preexisting renal dysfunction, hypertension, and diabetes are associated with coronary artery disease. The Society of Cardiovascular Computed Tomography guideline mentions that ICM volume of coronary CT angiography should be minimized, and lower tube voltage scans or VMI using multi-energy CT is recommended in patients at risk of PC-AKI [68]. Previous studies using DECT systems have shown that the low keV VMI provided equivalent to or better image quality for coronary CTA compared with conventional 100–120 kVp images even at 40–50% lower ICM doses [69–72] (Figs. 7 and 8). In state-of-the-art PCD-CT, Sartoretti et al. demonstrated that VMI at 40 keV was the optimal energy for achieving the highest iodine contrast and diagnostic confidence in coronary CTA, suggesting the potential for ICM dose reduction compared to EID-CT [73]. Subsequently, Emrich et al. showed that using VMI at 40 keV on a dual-source PCD-CT could reduce ICM concentration by 50% in a dynamic vessel phantom [74]. In a clinical investigation, Rajiah et al. indicated that high-pitch multi-energy coronary CTA could achieve diagnostic image quality while using 50% less ICM and 20% lower radiation doses with 50 keV VMI reconstruction in dual-source PCD-CT [75]. Cundari et al. also demonstrated that 45 keV VMI reconstruction with PCD-CT could reduce the ICM volume for CCTA by 40% while maintaining diagnostic image quality [76]. Beyond ICM dose reduction, calcium removal algorithms have been shown to improve the assessment of severely calcified coronary segments [77], while K-edge imaging enables non-invasive assessments of molecular features in vulnerable plaques [78], contributing to more appropriate patient management. As discussed later, window optimization is crucial for the accurate assessment of atherosclerotic lesions using low keV VMI.

### CT aortography

**Fig. 9:**
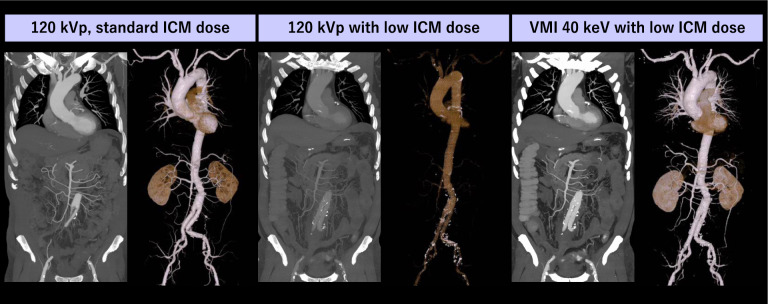
A 78-year-old man with a history of aortic replacement underwent follow-up CT scans at one-year intervals using both standard ICM dose (78 mL) and low ICM dose (25 mL) protocols on single-energy and dual-layer detector CT scanners, respectively. The standard image was reconstructed using conventional 120 kVp, while the low ICM dose images were reconstructed both with conventional 120 kVp and VMI at 40 keV. The reduction in arterial enhancement in the low ICM image was fully compensated by the VMI at 40 keV, resulting in even better arterial depiction compared to the standard ICM dose

**Fig. 10:**
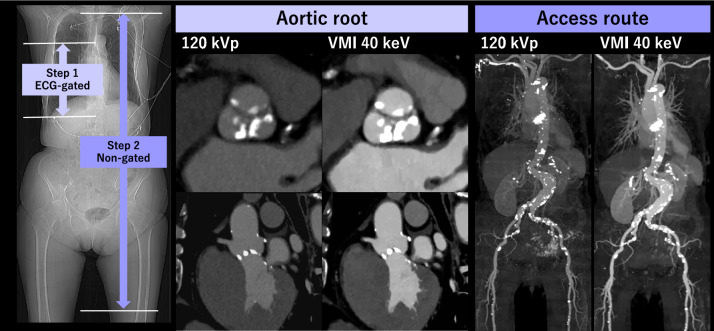
An 86-year-old woman with severe aortic valve stenosis and renal dysfunction underwent pre-TAVR CT using a two-step ICM injection protocol on dual-layer detector scanner. The first step involved ECG-gated scanning for aortic root with 14 mL of ICM, followed by non-gated scanning of the entire body with 15 mL of ICM. Using 40 keV VMI provided sufficient contrast enhancement for the pre-TAVR CT with a total of only 29 mL of ICM

CT aortography is the first-line imaging modality for the diagnosis and follow-up of aortic diseases such as aortic aneurysm and aortic dissection [79]. Similar to coronary artery disease, patients with aortic pathology often have comorbid conditions such as renal dysfunction, hypertension, and diabetes, which increases the risk of PC-AKI and necessitates ICM dose optimization. Previous studies on DECT have indicated that low keV VMI allowed for a reduction of ICM dose by up to 75% without degrading diagnostic image quality compared to conventional 120 kVp images [80, 81] (Fig. 9). Higashigaito et al. recently demonstrated that low keV VMI from a dual-source PCD-CT scanner enabled ICM dose reduction by 25% for thoracoabdominal CTA compared with low tube voltage scanning (median 90 kVp, range 70–100 kVp) performed with EID-CT, highlighting the added value of state-of-the-art multi-energy technologies [82]. Transcatheter aortic valve replacement (TAVR) planning requires ECG-gated aortic root CTA to determine the optimal size and type of prosthesis, as well as CTA scans to assess the access route through the aorta and pelvic arteries. Reducing the volume of ICM in TAVR planning CT is particularly important because most patients with aortic stenosis are elderly and suffer from renal dysfunction and severe heart failure [83]. In low-keV VMI from DECT, there are two approaches for ICM injection: 1) a two-step ICM bolus injection, with one bolus for ECG-gated CTA to assess the aortic root and another for non-ECG-gated CTA to assess the access route, or 2) a single ICM bolus injection covering the entire scan range from chest to pelvis with ECG-gated CTA. Previous studies have demonstrated the potential of low-keV VMI in both methods for reducing ICM dose while preserving diagnostic image quality [84, 85] (Fig. 10). The recently introduced dual-source PCD-CT allows high-pitch multi-energy acquisitions, which may further facilitate ICM dose reduction.

### Runoff lower extremity CTA

**Fig. 11:**
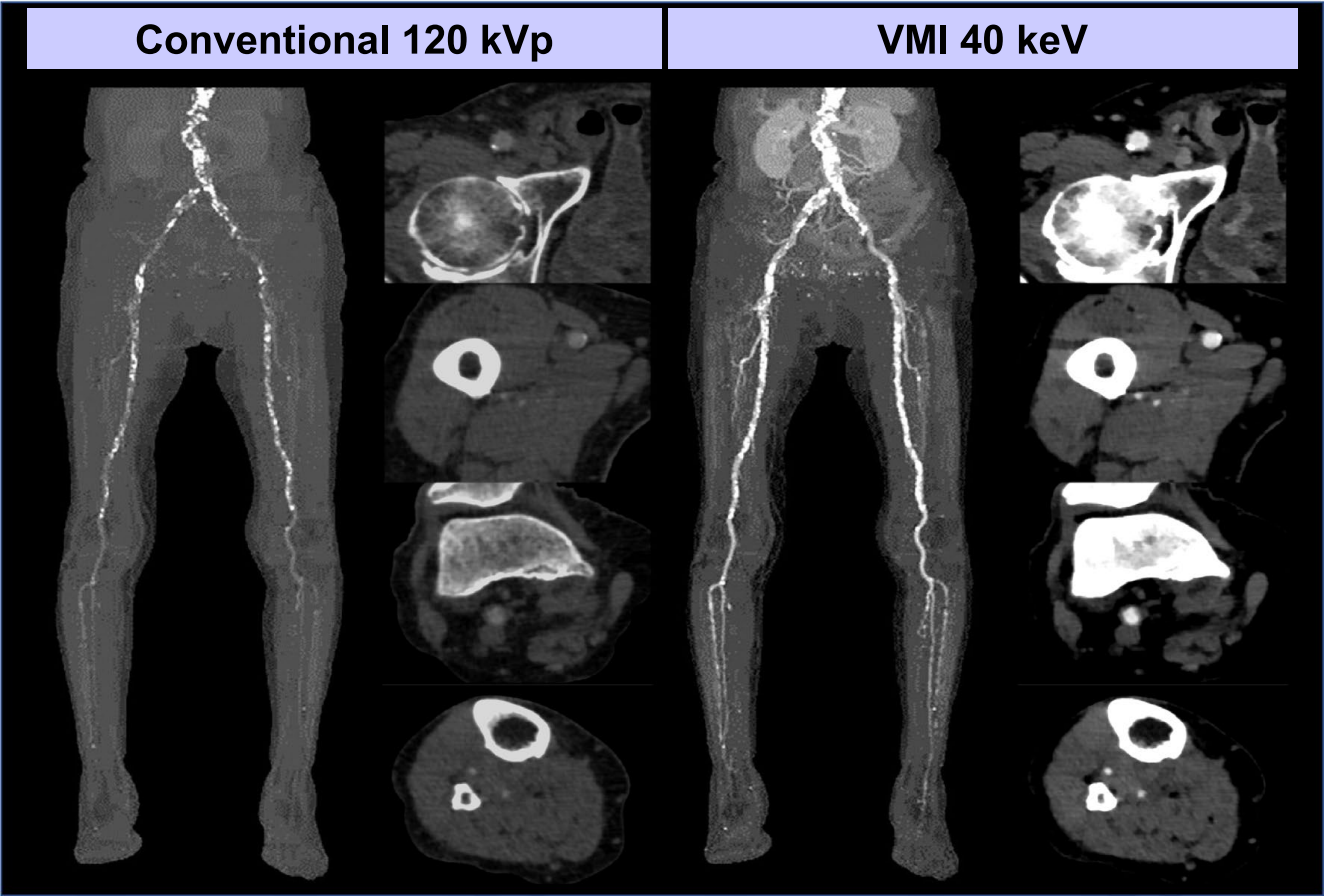
A 91-year-old woman with toe gangrene underwent runoff CTA using dual-layer detector scanner. A total of 25 mL of ICM (370 mgI/mL, diluted with 50 mL normal saline) was injected over 25 s. Conventional imaging at 120 kVp demonstrated insufficient arterial opacity, while VMI at 40 keV showed improved visibility of the lower extremity arteries

Peripheral artery disease (PAD), characterized by atherosclerotic stenosis or occlusion in the peripheral arteries of the lower extremities, is an important cause of death and disability worldwide [86]. Runoff CTA is a non-invasive examination for the diagnosis of PAD, but due to the long scanning range, relatively large volumes of iodine contrast media (ICM), up to 150 mL, with a long injection duration have been required [87]. Multi-energy CT-derived VMI in conjunction with optimized scanning timing provides sufficient and homogeneous enhancement for runoff CTA with a small amount of ICM. Researchers who used 40 keV VMI from dual-layer spectral detector CT, demonstrated that the ICM dose for runoff CTA can be reduced by 50% compared to the conventional 120 kVp protocol without degrading image quality or increasing radiation dose [88, 89] (Fig. 11). In the first generation dual-source PCD-CT, Rippel et al. demonstrated that 40–60 keV VMI yielded significantly higher SNR and CNR for runoff CTA than low tube voltage 80 kVp and 100 kVp scanning with EID CT, indicating its potential for greater ICM dose reduction capability [90].

### Abdominal CT

**Fig. 12:**
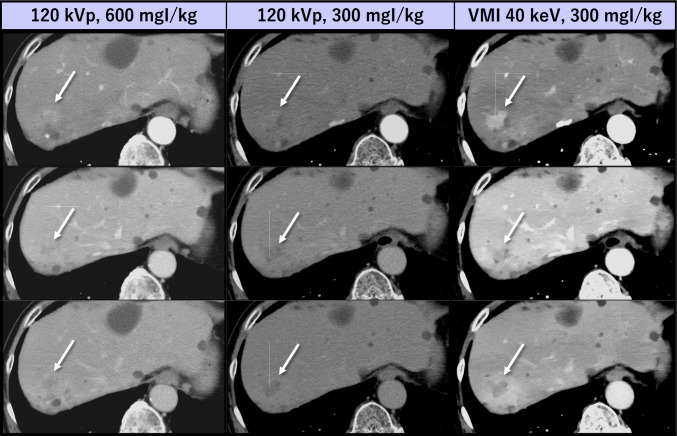
A 78-year-old man with hepatocellular carcinoma underwent multiphase hepatic CT with both standard (600 mgI/kg) and low (300 mgI/kg) ICM dose protocols on single-energy and dual-layer detector CT scanners, respectively, at 6 months interval. Compared with the standard protocol, better lesion conspicuity was attained at 40 keV VMI even with a 50% reduction in ICM dose without increasing radiation dose (size specific dose estimate: 15.4 vs. 14.7 mGy)

**Fig. 13:**
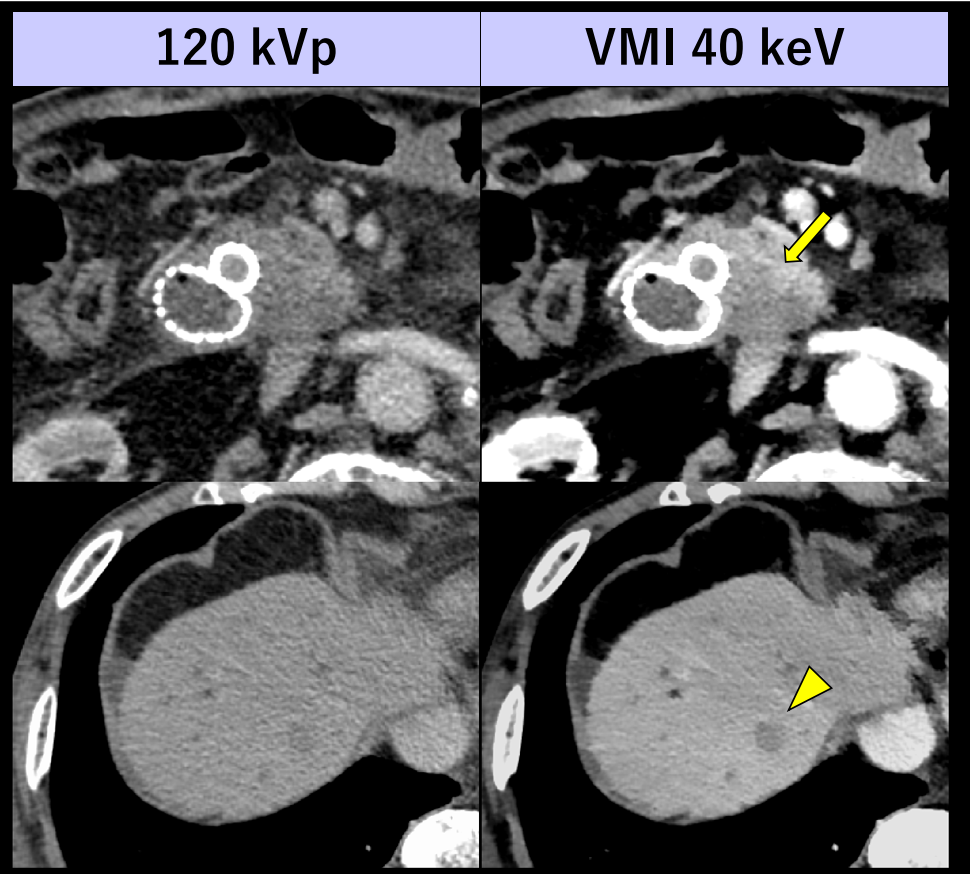
An 83-year-old man with pancreatic ductal adenocarcinoma in the pancreatic head and hepatic metastasis underwent multiphase pancreas CT (upper row: pancreatic phase; lower row: portal venous phase) using 33 mL of ICM (200 mgI/kg) on dual-layer detector scanner. In the 40 keV VMI, both the pancreatic and hepatic lesions are clearly delineated, in contrast to the conventional 120 kVp images where both lesions are barely visible due to insufficient contrast enhancement

**Fig. 14:**
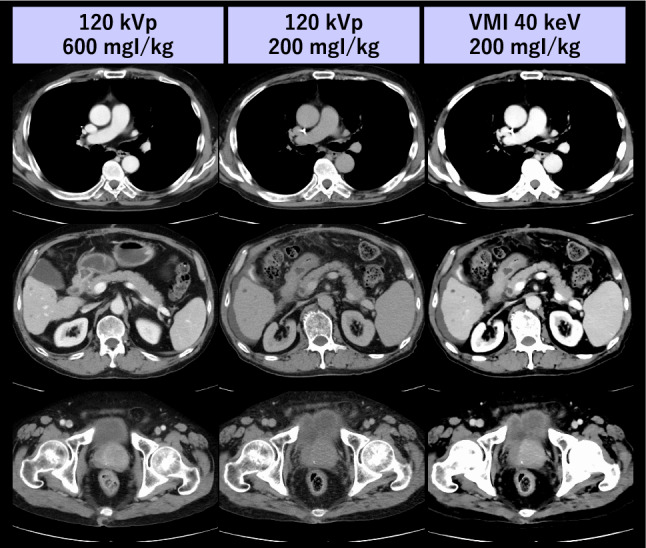
A 72-year-old man with esophageal cancer underwent scanning with both a standard 120 kVp protocol (iodine dose, 37 g; SSDE, 19.2 mGy) and a low ICM dose CT protocol (iodine dose, 12.6 g; SSDE, 16.3 mGy) on single-energy and dual-layer detector CT scanners, respectively, with a 12-month interval. Compared with the 120 kVp images, an equivalent or higher iodine contrast was attained at VMI 40 keV, despite a 66% reduction in iodine dose

For appropriate detection, characterization, and staging of abdominal organ tumors, administration of a relatively large amount of ICM and multiphasic acquisitions involving high radiation exposure are usually required, necessitating the optimization of both ICM and radiation doses to mitigate potential adverse effects. Several investigations on multiphase hepatic CT have demonstrated that lesion conspicuity can be improved with low-keV VMI from DECT, allowing for simultaneous reduction in radiation and ICM doses while preserving or even improving hepatic tumor conspicuity [91, 92] (Fig. 12). For multiphase pancreatic CT, low-keV VMI improves tumor-to-pancreas contrast and evaluation for vessel involvement compared to conventional 120 kVp [93, 94] images, enabling comprehensive evaluation of pancreatic tumors even with a substantially reduced ICM dose (Fig. 13). Similarly, previous reports have indicated that the ICM dose for chest-abdomen-pelvis CT performed for screening of malignancy, trauma, or inflammatory processes can be reduced by at least 50% while preserving diagnostic image quality at a lower radiation dose, as long as image noise is adequately reduced for the lowest energy VMI [95, 96] (Fig. 14).

**Fig. 15:**
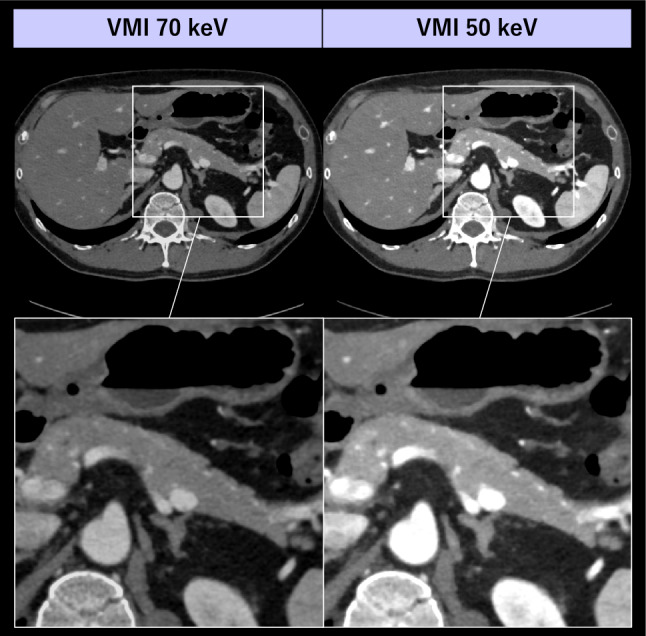
A 40-year-old man underwent abdominal CT with a low ICM dose protocol (350 mgI/kg) on a PCD-CT scanner. Compared with the VMI 70 keV images, iodine contrast of VMI 50 keV is considerably improved while preserving diagnostically acceptable image noise level. Image courtesy of Medical Scanning Tokyo

With recently introduced PCD-CT, Higashigaito et al. suggested that 50 keV is the optimal VMI energy for portal-phase abdominal CT [97]. Decker et al. also found that low-keV VMI (< 70 keV) from PCD-CT significantly improved the conspicuity of pancreatic ductal adenocarcinomas compared to EID-CT [94]. Moreover, recent studies have demonstrated that 50 keV VMI enabled a 50% reduction in ICM dose without compromising image quality in abdominal CT [98, 99]. These reports highlight the potential of PCD-CT for optimizing ICM dose in parenchymal organ assessments (Fig. 15).

## Current issues and related topics

### Alteration of iodine density

The iodine density obtained with multi-energy CT has been proposed as a quantitative parameter for tumor and tissue characterization, perfusion assessments, and the prediction of treatment responses and patient prognoses [100–104]. In a reduced ICM dose setting, the iodine density at the lesions and structures of interest can be lower relative to the standard ICM dose, meaning that the diagnostic/predictive values and cutoff values shown in previous investigations cannot be directly applicable. Iodine density normalization with blood pools such as the aorta may be a possible approach to address inter-protocol variability [105], but quantification consistency between standard and reduced ICM dose settings needs to be investigated.

### Accentuated metal artifact

**Fig. 16:**
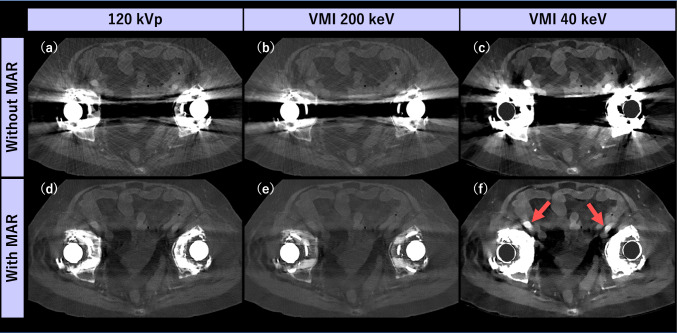
A 68-year-old woman with bilateral total hip replacement who underwent contrast-enhance CT using dual-layer detector scanner. Compared to conventional 120 kVp (**a**), VMI at 200 keV without MAR (**b**) shows limited artifact reduction and poor visualization of iodinated structures. In contrast, MAR-processed images (**d**–**f**) show significant reduction in metallic artifacts. The combination of 200 keV with MAR (**e**) is suitable for evaluating bone structures but offers no advantage for soft tissue assessment compared to 120 kVp with MAR (**d**). The best visualization of iodinated structure such as arteries (arrows) is obtained at VMI 40 keV with MAR (**f**)

At lower keV settings, metal artifacts due to photon starvation or beam hardening are substantially accentuated compared to conventional 120 kVp images. High-keV VMI can reduce the visibility of mild beam hardening artifacts from low-density metal, but it compromises iodine contrast, making it unsuitable for low ICM dose settings. Metal artifact reduction (MAR) algorithms are superior for reducing strong artifact due to photon starvation from high-density metal (e.g., bilateral total hip replacement) and preserving soft tissue contrast compared to high-keV VMI. Given that the current commercially available multi-energy scanners allow simultaneous utilization of MAR and VMI [106–111], combining low-keV VMI with MAR may be suitable for assessing iodinated structures adjacent to metallic implants that are not adequately visible on conventional 120-kVp images (Fig. 16).

### Window optimization for the evaluation of calcified plaque

**Fig. 17:**
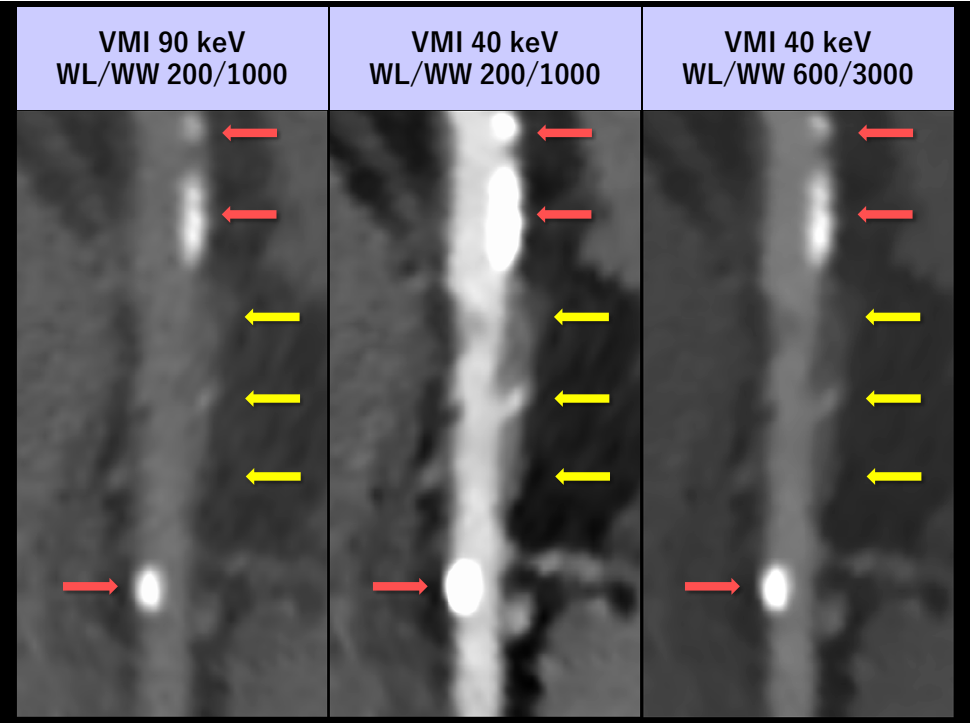
Stretched curved MPR of the right coronary artery in an 86-year-old man who underwent coronary CTA using dual-layer detector scanner with a low ICM dose (18 mL, 370 mgI/mL). The images were reconstructed with VMI at 90 and 40 keV. At the same window setting (WL/WW: 200/1000 HU), blooming artifacts from calcified plaques lead to an overestimation of luminal stenosis in the 40 keV VMI compared to the 90 keV VMI (red). However, mixed plaques with positive remodeling (yellow) are better visualized in the 40 keV VMI due to decreased attenuation of pericoronary fat and increased luminal attenuation. By optimizing the window setting for 40 keV, the degree of visual blooming artifacts from calcified plaques remains consistent with those at 90 keV, while luminal brightness, sharpness, and depiction of mixed plaques with positive remodeling are superior in the 40 keV images compared to the 90 keV images

The window setting of low-keV VMI should be optimized according to the evaluated objects, which is particularly important for accurately assessing atherosclerotic lesions [112]. When the window setting is fixed at entire energy range, low-keV VMI visually pronounced blooming artifacts from calcified plaque and can lead to overestimation of the luminal stenosis. In this context, prior studies have shown that VMI at around 90 keV is the best energy for evaluating calcified plaque [113, 114]. However, our experience suggests that optimizing the window display may help preserve the visual appearance and quantitative luminal and calcified sizes even at the lowest keV levels (Fig. 17). Notably, Yunaga et al. demonstrated that the high keV VMI (80–140 keV) did not reduce blooming artifact and improve the visual conspicuity of lumen [115], which may be particularly relevant for ICM dose reduction protocols. As the current evidence remains limited, the impact of VMI energy level and window settings on the evaluation of luminal stenosis in the presence of calcified plaques warrants further investigation.

### Effect of iodine concentration on arterial enhancement

In CTA, total iodine dose (mgI) and iodine delivery rate (mgI/s) are main parameters in optimizing arterial attenuation [116]. The high concentrations of ICM have been used for traditional CTA protocols due to the effective delivery of iodine in a short duration, but the optimal iodine concentration needs to be adopted to the new standard for lower energy settings. When the iodine delivery rate and total iodine dose are kept constant, a low concentration or diluted ICM provides equivalent or better arterial enhancement compared with a high ICM concentration [117, 118]. Because of the lower viscosity and lower injection pressure, using a low concentration or diluted ICM may be desirable, especially for patients with tiny/fragile veins and without comorbidities susceptible to volume overload [117, 119]. The representative trigger thresholds in bolus-tracking techniques for CTA are typically 100–150 HU when monitoring scans are performed at 120 kVp, whereas the threshold for multi-energy CT can be adjusted depending on the targeted keV settings due to improved iodine contrast. Noda et al. recently demonstrated that lowering the bolus-tracking threshold from 100 to 30 HU for a 40 keV VMI resulted in improved arterial enhancement compared to SECT, even with a 50% reduction in ICM dose, highlighting the potential importance of tailored threshold optimization for effective ICM dose reduction [120].

### Current limitations and future directions of photon-counting detector CT

As previously described, PCD-CT is expected to offer superior image quality and dose efficiency for ICM dose reduction compared to conventional EID-CT [22, 23, 25, 26]. However, current limitations of PCD-CT include high cost, limited availability, and the fact that only a single vendor currently provides commercialized systems, which are restricted to using cadmium telluride (CdTe) as the detector material. Future developments may involve the clinical adoption of alternative detector types such as cadmium zinc telluride (CZT) or silicon-based sensors, as well as systems with spectral resolution enabling k-edge imaging. Further research is warranted to evaluate the potential of these configurations, including determining to what extent each multi-energy CT system can reduce the required iodine dose while preserving image quality and diagnostic performance.

## Conclusion

Low keV VMI derived from multi-energy CT systems is promising technique for optimizing the ICM dose, which can mitigate the possible concerns for patient safety, increased medical cost, and environmental negative impacts. As outlined in this article, multi-energy CT enables a reduction in ICM dose for most clinical indications, while preserving or even improving image quality compared with conventional SECT scanning.
